# Relative efficacy of antibody-drug conjugates and other anti-HER2 treatments on survival in HER2-positive advanced breast cancer: a systematic review and meta-analysis

**DOI:** 10.1186/s12885-024-12478-1

**Published:** 2024-06-08

**Authors:** Zian Kang, Yuqing Jin, Huihui Yu, Su Li, Yingjie Qi

**Affiliations:** 1 Department of Pharmacy, Cancer Hospital of China Medical University, Liaoning Cancer Hospital & Institute, Shenyang, Liaoning Province 110042 China; 2 School of Life Science and Biopharmaceutics, Shenyang Pharmaceutical University, Shenyang, China; 3 Department of Cancer Prevention and Control, Cancer Hospital of China Medical University, Liaoning Cancer Hospital & Institute, Shenyang, China

**Keywords:** Antibody-drug conjugates, Breast cancer, Overall survival, Progression-free survival, Meta-analysis

## Abstract

**Background:**

Novel antibody-drug conjugates (ADCs) drugs present a promising anti-cancer treatment, although survival benefits for HER2-positive advanced breast cancer (BC) remain controversial. The aim of this meta-analysis was to evaluate the comparative effect of ADCs and other anti-HER2 therapy on progression-free survival (PFS) and overall survival (OS) for treatment of HER2-positive locally advanced or metastatic BC.

**Methods:**

Relevant randomized controlled trials (RCTs) were retrieved from five databases. The risk of bias was assessed with the Cochrane Collaboration’s tool for RCTs by RevMan5.4 software. The hazard ratio (HR) and 95% confidence intervals (CIs) were extracted to evaluate the benefit of ADCs on PFS and OS in HER2-positive advanced BC by meta-analysis.

**Results:**

Meta-analysis of six RCTs with 3870 patients revealed that ADCs significantly improved PFS (HR: 0.63, 95% CI: 0.49–0.80, *P* = 0.0002) and OS (HR: 0.79, 95% CI: 0.72–0.86, *P* < 0.0001) of patients with HER2-positive locally advanced or metastatic BC. Subgroup analysis showed that PFS and OS were obviously prolonged for patients who previously received HER2-targeted therapy. Sensitivity analysis and publication bias suggested that the results were stable and reliable.

**Conclusion:**

Statistically significant benefits for PFS and OS were observed with ADCs in HER2-positive locally advanced or metastatic BC, especially for those who received prior anti-HER2 treatment.

**Supplementary Information:**

The online version contains supplementary material available at 10.1186/s12885-024-12478-1.

## Introduction

Breast cancer (BC) is a leading cause of cancer-related morbidity and mortality of women aged 20–59 years [1, 2]. Human epidermal growth factor receptor-2 (HER2) is overexpressed in about 20–25% of BC and closely correlated to the proliferative and invasive capabilities of BC cells [3]. The biological behavior is characterized by high recurrence, metastasis, drug resistance and poor prognosis [4]. Conventional treatments include large molecule monoclonal antibodies, small molecule tyrosine kinase inhibitors and cytotoxic chemotherapy regimens. However, these modalities often fall short due to their limited efficacy or serious adverse drug reactions [5–7]. Thus, more effective and safer treatment strategies are urgently needed [8].

Antibody-drug conjugates (ADCs) targeting HER2 gene amplification or protein overexpression can greatly improve the prognosis of patients with HER2-positive locally advanced or metastatic BC. ADCs are composed of a monoclonal antibody, cytotoxic agent and chemical linker. When the monoclonal antibody omponent of the ADC complex is endocytosed, the linker is cleaved by the low pH of lysosomes, which releases the cytotoxic agent to cause damage to DNA or microtubule proteins, resulting in death of tumor cells [9–11]. Therefore, ADCs has the capacity for precise targeting, stable therapeutic efficacy and low toxicity [12, 13].

Numerous clinical trials have investigated the efficacy of ADCs targeting HER2 for treatment of HER2-positive locally advanced or metastatic BC. In terms of PFS, the international multicenter phase III clinical EMILIA trial [14, 15] validated that trastuzumab emtansine (T-DM1) significantly improved (*p* < 0.001) progression-free survival (PFS) of patients with HER2-positive advanced BC. Notably, MARIANNE study [16] revealed that T-DM1 did not bring obvious PFS benefits compared to other anti-HER2 treatments. In terms of OS, the TH3RESA study [17, 18] underscored the median OS was significantly prolonged in T-DM1 group. However, MARIANNE study found that the OS of the intervention group containing T-DM1 was not inferior to that of trastuzumab combined with taxanes.

In consideration of the conflicting outcomes of previous individual clinical trial, the aims of this systematic review and meta-analysis were to explore the efficacy of ADCs on PFS and OS for treatment of HER2-positive locally advanced and metastatic BC and identify patients who would most benefit from treatment.

## Materials and methods

This systematic review and meta-analysis followed the principle of the Preferred Reporting Items for Systematic Reviews and Meta-Analyses (PRISMA) [19]. The protocol was registered in PROSPERO (CRD42023477508).

### Search strategy

Relevant clinical studies of the efficacy of ADCs targeting HER2-positive advanced BC were retrieved from the PubMed, Web of Science, Cochrane Library, Embase, and Clinical Trials databases (from inception to March 2023). The main therapy-related search terms were (human epidermal growth factor receptor 2 OR HER-2 protein OR EGFR2 protein) AND (Antibody-Drug Conjugates OR ADCs OR ADC) AND (survival OR overall survival OR OS OR progression-free survival OR PFS). The disease-related search term was breast cancer OR breast neoplasm OR breast tumor. Supplementary File. 1 described the detailed methods used to search. Also, the reference sections of all articles were manually searched to identify other eligible studies.

### Eligibility criteria

The eligibility criteria of prospective studies included (1) randomized controlled trials (RCTs) of patients with HER2-positive locally advanced or metastatic BC; (2) immunohistochemistry analysis (with 3+) and/or fluorescence in situ hybridization (amplification ratio ≥ 2.0) of HER2-positive; (3) intervention with ADCs; and (4) inclusion of PFS or OS with the hazard ratio (HR) and 95% confidence intervals (95% CIs). The exclusion criteria were (1) early stage BC; (2) non-RCTs, HER2-negative, animal studies, reviews, and case reports; (3) failure to report PFS or OS.

### Study selection and data extraction

Two of the authors (KZA and QYJ) independently screened the articles based on the eligibility criteria and extracted the data. If no consensus was reached, a third author (LS) made the final decision to either include or exclude the study.

The selection method were as follows: (1) P: patients in the original research were diagnosed with HER2-positive advanced breast cancer; (2) I: HER2-positive breast cancer patients received anti-HER2 treatment of ADC; (3) C: HER2-positive breast cancer patients received other anti-HER2 treatment; (4) O: studies that reported HR and 95% CIs of PFS or OS; and (5) S: RCTs.

The extracted data included the name of the trial, name of the first author, publication year, study phase, study population, study design, intervention, sample size, HRs, and 95% CIs for PFS and OS. The extraction data were recorded in an Excel file (Microsoft Corporation, Redmond, WA, USA).

### Quality assessment

The risk of bias was assessed in accordance with the criteria in the Cochrane Handbook for Systematic Reviews of Interventions. The quality of the included studies was evaluated with Cochrane Collaboration’s “Risk of Bias” for RCTs by the Review Manager 5.4 software [20, 21]. Two of the authors independently performed the risk of bias evaluation from seven aspects: random sequence generation, allocation concealment, blinding for participants and personnel, blinding for outcome assessment, incomplete outcome data, selective reporting and other bias. The risk of bias was graded as low, high, or unclear.

### Statistical analysis

All statistical analyses were performed with RevMan5.4 and STATA 16.0 software (StataCorp LLC, College Station, TX, USA). The HRs and 95% CIs of each study were pooled to evaluate the efficacy of ADCs as compared to other HER2-targeting regimens in HER2-positive advanced BC. The heterogeneity among studies was assessed using Cochrane’s Q and *I*^*2*^ statistics. A probability (*P*) value ≤ 0.10 or *I*^*2*^ value ≥ 50% indicated significant heterogeneity, thus a random-effects model was selected; Otherwise, a fixed-effects model was adopted.

In addition, subgroup analysis was conducted to identify the origin of heterogeneity. Sensitivity analysis was performed to determine the reliability of the results. Egger’s test was conducted to assess publication bias. The publication bias was absent if *P* > 0.05 in Egger’s test [22].

## Results

### Study selection and characteristics

**Fig. 1:**
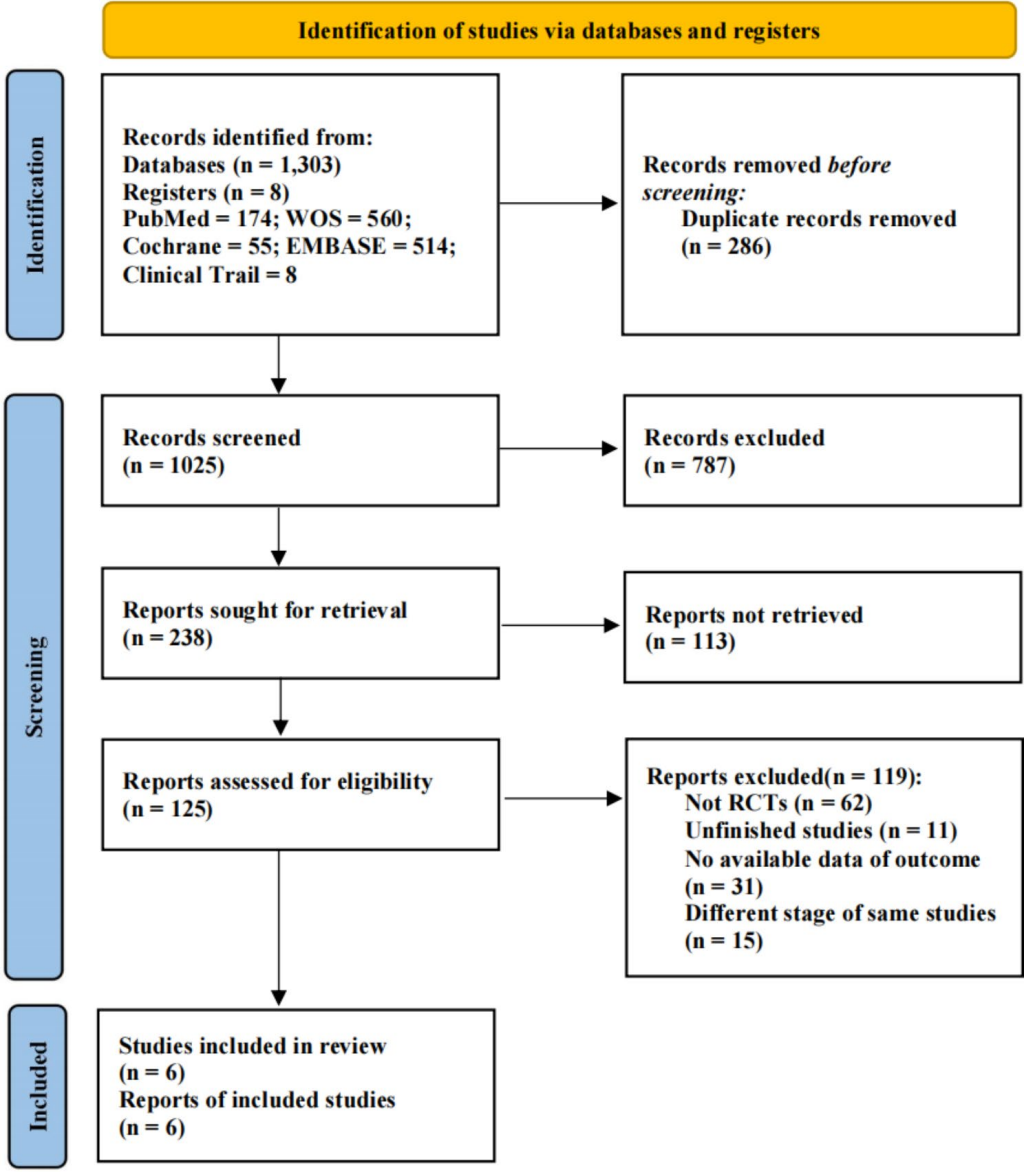
Flow chart of the literature search and study selection

In total, 1311 articles were retrieved. Of these, 286 duplicated studies were excluded. After reviewing the titles and abstracts, an additional 900 studies, which included reviews, commentaries, case reports, and irrelevant studies, were also excluded. Subsequently, the full text of 125 studies was examined. Finally, six studies (7 RCTs) were included in the meta-analysis [15–17, 23–25]. A flow chart of the study selection process is presented in Fig. 1.

**Fig. 2:**
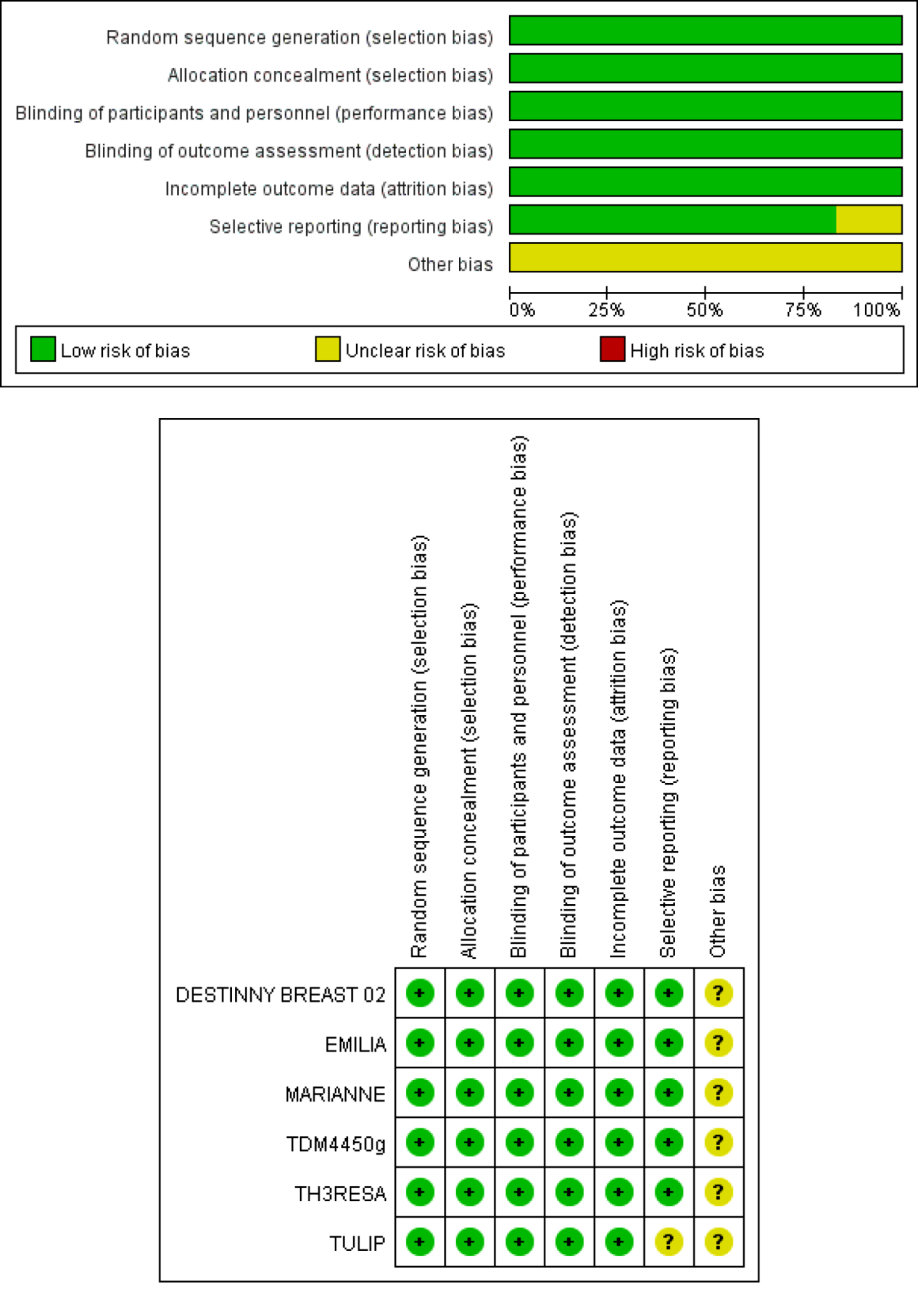
Quality assessment for risk of bias for the included randomized controlled trials

The six studies included 3870 patients with HER2-positive advanced BC, were published between 2013 and 2023, and included one phase II and five phase III studies. Five studies included two groups and one included three groups. The ADCs used in the studies included T-DM1, trastuzumab duocarmycin (SYD985), and trastuzumab deruxtecan (T-Dxd). All studies compared the efficacy of ADCs versus other HER2-targeting regimens. The characteristics of the selected studies are presented in Table 1 and the quality evaluation results are shown in Fig. 2.

**Table 1:** Characteristics of the included studies in the meta-analysis

Study	First author	Year	Phase	Population	Study design	Intervention	Population(*n*) intervention/total(%)	PFS HR (95%CI)	OS HR (95%CI)
TDM4450g	Hurvitz [23]	2013	II	Stage III～IV, LABC/MBC	Multicenter RCT	T-DM1	67(48.91%)	0.59(0.36,0.97)	1.06(0.48,2.35)
EMILIA	Verma [15]	2017	III	Stage III#xFF5E;IV, LABC/MBC	Multicenter RCT	T-DM1	495(49.95%)	0.65(0.55,0.77)	0.75(0.64,0.88)
MARIANNE(A)	Perez [16]	2017	III	Stage III#xFF5E;IV, LABC/MBC	Multicenter RCT	T-DM1	367(33.45%)	0.91(0.75,1.10)	0.93(0.75,1.16)
MARIANNE(B)	Perez [16]	2017	III	Stage III#xFF5E;IV, LABC/MBC	Multicenter RCT	T-DM1 + Pertuzumab	363(33.09%)	0.87(0.71,1.06)	0.86(0.69,1.08)
TH3RESA	Krop [17]	2017	III	Stage III#xFF5E;IV, LABC/MBC	Multicenter RCT	T-DM1	404(67.11%)	0.53(0.42,0.66)	0.68(0.54,0.85)
TULIP	Manich [24]	2021	III	Stage III#xFF5E;IV, LABC/MBC	Multicenter RCT	SYD985	291(66.59%)	0.64(0.49,0.84)	0.87(0.68,1.11)
DESTINY BREAST 02	Fabrice [25]	2023	III	Stage IV, MBC	Multicenter RCT	T-DXd	406(66.78%)	0.36(0.28,0.45)	0.66(0.50,0.86)

LABC, locally advanced breast cancer; MBC, metastatic breast cancer; RCT, randomized clinical trial; TDM-1, trastuzumab emtansine; T-DXd, Trastuzumab deruxtecan; SYD985, trastuzumab duocarmazine; PFS, progression free survival; OS, overall survival; HR, hazard ratio; 95% CI, 95% confidence interval

### Meta‑analysis of PFS

**Fig. 3:**
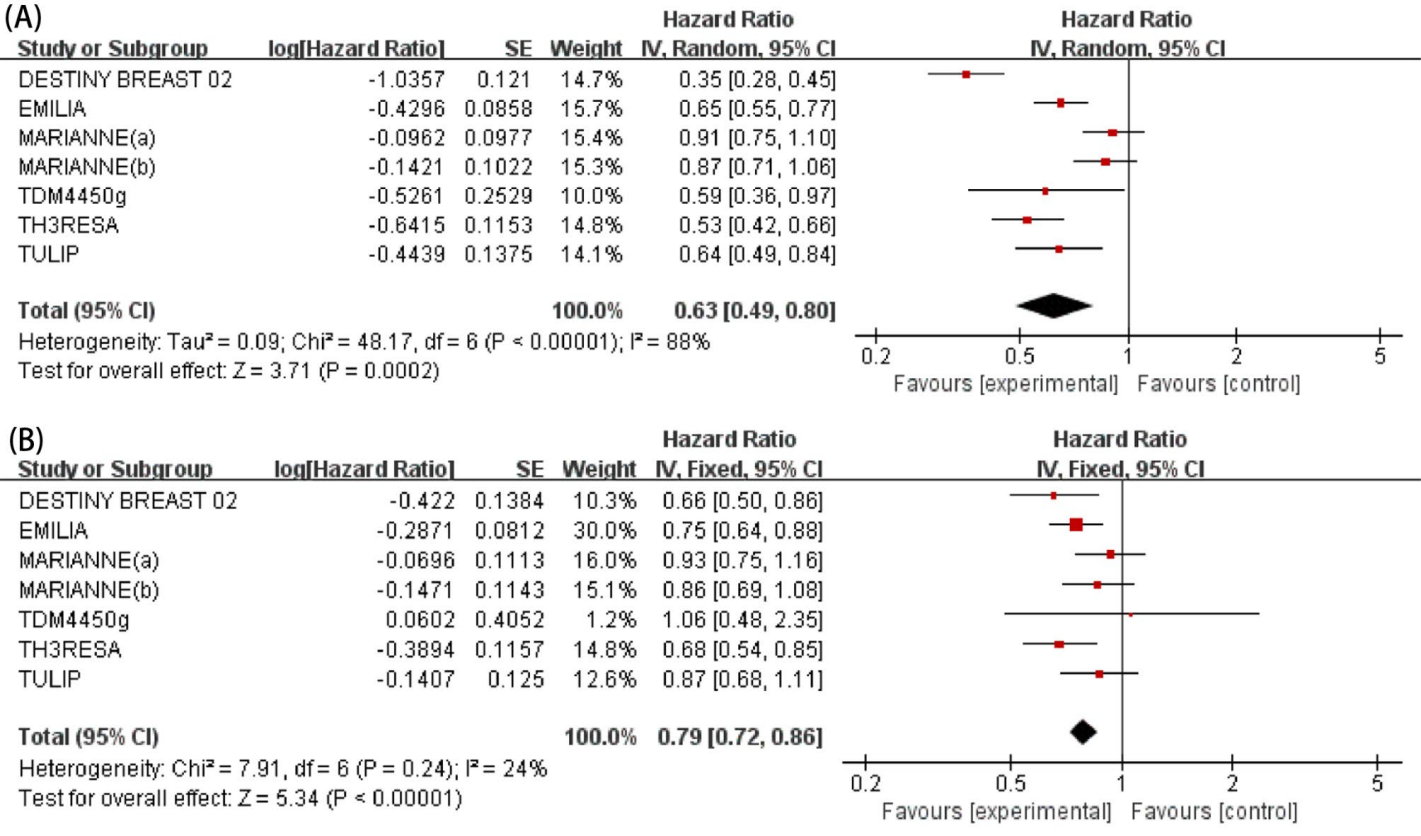
Forest plots of HR for PFS (**A**) and HR for OS (**B**) in patients with ADCs versus other HER2-targeted regimens

**Fig. 4:**
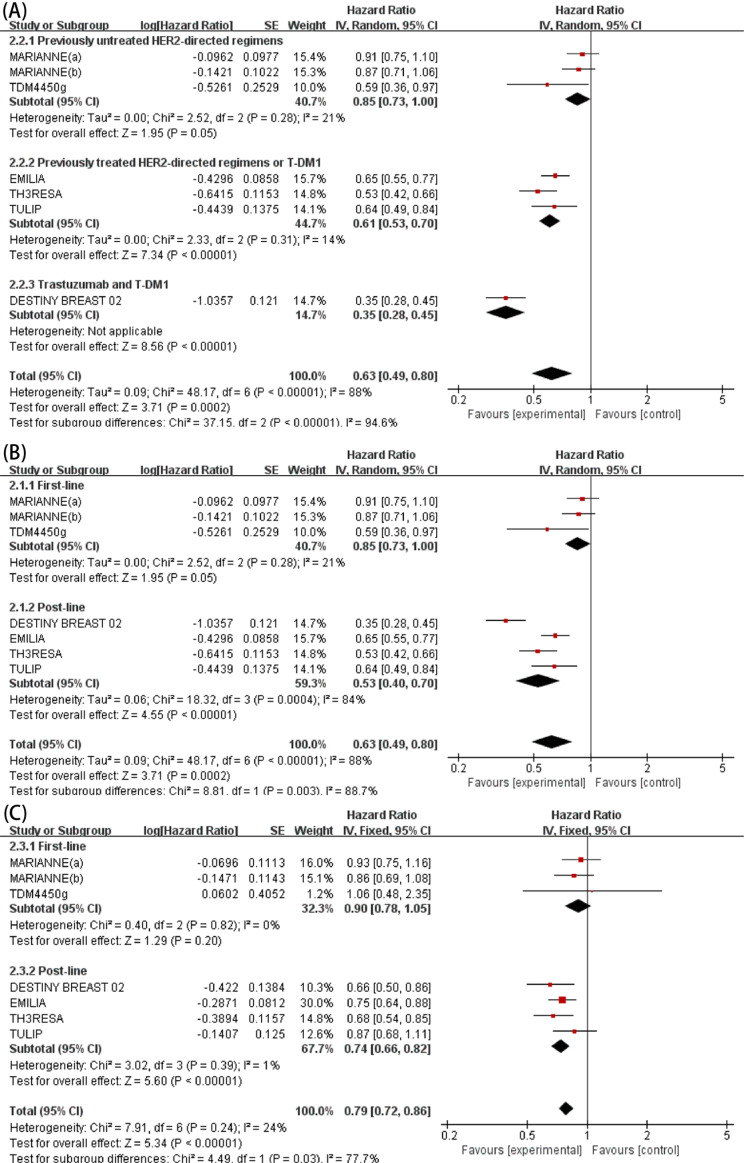
Subgroup analysis for PFS by prior anti-HER2 treatment categories in patients with HER2-positive advanced breast cancer (**A**). Subgroup analysis for PFS by treatment line in patients with HER2-positive advanced breast cancer (**B**). Subgroup analysis for OS by treatment line in patients with HER2-positive advanced breast cancer (**C**)

All six studies (7 RCTs) reported the efficacy of ADCs versus other HER2-targeted regimens for PFS of patients with HER2-positive locally advanced or metastatic BC. Because of the presence of heterogeneity among studies (*I*^*2*^ = 88%, *P* < 0.00001), a random-effects model was adopted for analysis. The pooled HR indicated that ADCs had significantly improved PFS as compared to other HER2-targeting drugs plus chemotherapy (HR: 0.63, 95% CI: 0.49–0.80, *P* = 0.0002; Fig. 3). Subgroup analysis stratified by prior anti-HER2 treatment was performed to further explore the potential origin of heterogeneity. Among patients who did not receive anti-HER2 therapy, there was no significant difference in outcomes between ADCs and other HER2-targeted regimens plus chemotherapy (HR: 0.85, 95% CI: 0.73-1.00, *P* = 0.05; Fig. 4), while additional treatment with ADCs conveyed a significant benefit to patients who previously received HER2-targeted therapy (HR: 0.61, 95% CI: 0.53–0.70, *P* < 0.00001; Fig. 4) or trastuzumab and T-DM1 therapy (HR: 0.35, 95% CI: 0.28–0.45, *P* < 0.00001; Fig. 4). However, subgroup analysis stratified by treatment lines did not reduce heterogeneity (Fig. 4).

### Meta‑analysis of OS

All six studies (7 RCTs) assessed the OS of patients with HER2-positive locally advanced or metastatic BC who received ADCs versus other HER2-targeted regimens.

Due to the absence of heterogeneity among studies (*I*^*2*^ = 24%, *P* = 0.24), a fixed-effects model was used for analysis. The pooled HR revealed that ADCs prolonged OS of patients with HER2-positive advanced BC (HR: 0.79, 95% CI: 0.72–0.86, *P* < 0.0001; Fig. 3). Subgroup analysis stratified by treatment line was performed to identify patients who would most benefit from treatment with ADCs. The results found that ADCs did not significantly improve OS as first-line therapy (HR: 0.90, 95% CI: 0.78–1.05, *P* = 0.82; Fig. 4). However, ADCs as post-line therapy significantly improved OS (HR: 0.74, 95% CI: 0.66–0.82, *P* = 0.39; Fig. 4).

### Sensitivity analysis and publication bias

**Fig. 5:**
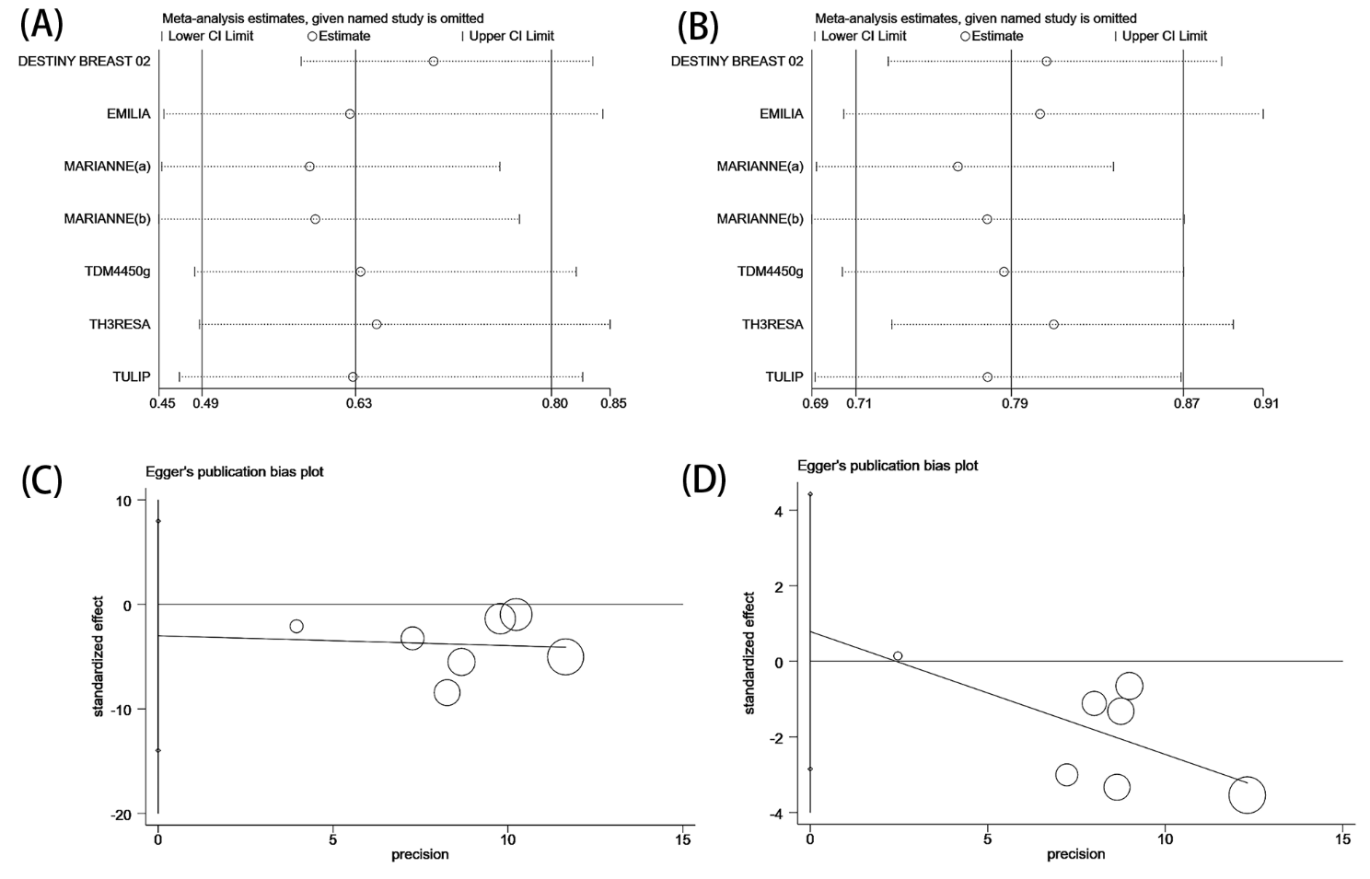
Sensitivity analysis for PFS (**A**) and for OS (**B**). Egger’s test of publication bias for PFS (**C**) and for OS (**D**)

Sensitivity analysis performed by removing each study showed that the results of the meta-analysis were reliable (Fig. 5). The Egger’s test results indicated the absence of publication bias in regard to PFS and OS (PFS: *P* = 0.513, OS: *P* = 0.602; Fig. 5).

## Discussion

HER2 was identified as a critical prognostic factor in BC. Overexpression of the HER2 gene, which stimulated transphosphorylation of the HER2 protein on the cell membrane and continuous activation of downstream signal transduction pathways related to proliferation of tumor cells, was predictive of high malignancy, rapid progression, short chemotherapy remission, and poor overall survival [26, 27]. ADCs targeting HER2 have become an emerging treatment strategy for HER2-positive advanced BC. Although various international multicenter clinical trials investigated the efficacy of ADC vs. other anti-HER2 drugs, the results were often contradictory. Therefore, the aim of this systematic review and meta-analysis of seven RCTs was to assess the efficacy of ADCs for HER2-positive advanced BC and to identify patients who would most benefit from treatment.

Our results showed a statistically significant prolongation of PFS and OS in patients treated with ADCs. The potential mechanism might be related to structure and function of ADCs. ADCs combined with conventional chemotherapy drugs with highly selective targeted monoclonal antibody not only inhibited HER2 dimerization and activation of downstream signaling pathways, but also promoted internalization of the ADC complex. Targeting of HER2 positive tumor cells increased drug selectivity and cytotoxic effects in tumor cells, while reducing adverse effects resulting from systemic drug exposure [28–30]. The unique pharmacokinetic and pharmacodynamic characteristics of ADCs can improve the outcomes of patients with HER2-positive locally advanced and metastatic BC.

Subgroup analysis showed that ADCs as a first-line treatment did not significantly improve PFS and OS of patients who did not receive prior anti-HER2 therapy. For these patients, high sensitivity to both ADCs and other HER2-targeted therapies plus chemotherapy could be attributed to receive drug treatment for the first time. Additionally, the dosage of chemotherapy drugs in other HER2-targeted therapies combined with chemotherapy regimens was much higher than for ADCs [31]. The anti-tumor effects of chemotherapeutic drugs was relatively pronounced, especially in the initial stage of treatment [32, 33], which might account for the lack of clear benefits for patients receiving ADCs vs. other HER2-targeted therapies plus chemotherapy.

Both PFS and OS were significantly improved among patients who previously received HER2-targeted therapies or T-DM1, possibly due to a greater tumor burden, severe systemic toxicity, lower patient tolerance and the emergence of drug resistance [34], which limited benefits of cytotoxic chemotherapy regimens. Therefore, ADCs with lower systemic toxicity and more accurate targeting might be more suitable for these patients. Besides, mutations to the α-subunit catalyzed by phosphatidylinositol-3-kinase or the loss of PTEN expression could lead to poor efficacy of trastuzumab or lapatinib. The results of the EMILIA and TH3RESA studies [15, 24] revealed that T-DM1 was therapeutically effective for cases with mutations to the α-subunit catalyzed by phosphatidylinositol-3-kinase, which potentially contributed to improve the PFS of patients treated with ADCs. Additionally, in the phase III TULIP study, some patients who received T-DM1 included in the ADCs group still achieved good clinical outcomes, which might be related to the intervention drug-SYD985, which is prodrug conjugated with trastuzumab via a cleavable linker. The unique design of the cleavable linker not only ensures high stability of ADCs in circulation, but also effective release of cytotoxic drugs in tumors via a bystander effect. The active SYD985 was released in tumor cells and resulting in DNA alkylation [35]. The activity of SYD985 is reportedly 53.7-and 2.8-fold greater than T-DM1 in vitro and in vivo, respectively [36]. Collectively, these factors likely contribute to the superior efficacy of ADCs.

For patients previously treated with trastuzumab and T-DM1 in advanced settings, PFS was significantly improved for those who received T-Dxd. T-Dxd is a new generation of ADCs with significantly superior efficacy as compared to traditional drugs owing to an improved structural design and mechanism of action. First, T-Dxd coupled of trastuzumab with the highly active topoisomerase I inhibitor Dxd through stable cleavable linker. The drug antibody ratio was 8, significantly higher than the drug antibody ratio of 2–4 of traditional ADCs. In addition, the anti-tumor activities of Dxd is 10-foled greater than SN38 (an active metabolite of irinotecan) and approximately 1000-fold greater than traditional chemotherapy drugs. Meanwhile, the anti-tumor mechanism of T-Dxd was different from that of microtubule chemotherapy drugs for breast cancer, which effectively avoided cross resistance. Moreover, T-Dxd exhibited excellent cell membrane permeability, allowing penetration of adjacent tumor cells to exert a strong bystander effect. Therefore, T-Dxd can achieve superior anti-tumor effects, even against tumors with low HER2 expression [37, 38].

This meta-analysis of seven RCTs was the first comprehensive study to compare the effectiveness of ADCs with other HER2-targeted therapies for treatment of HER2-positive advanced BC. Seven RCTs of 3870 patients tested three types of ADCs (T-DM1, SYD985, and T-Dxd). Notably, the global multicenter phase III clinical trials DESTINY BREAST 02 and TULIP (reported in 2023 and 2021, respectively) were not included in previous meta-analyses [39–41]. Comparatively, the present meta-analysis included high-quality trials with relatively larger cohorts and more available drugs, thus offering more reliable conclusions. Furthermore, subgroup analysis was performed to identify potential sources of heterogeneity. The results of the subgroup analysis were basically consistent with the preliminary analysis. These findings offer useful information for treatment of HER2-positive advanced BC.

There were some limitations to this meta-analysis that should be addressed. First, only six RCTs were included in this analysis, which excluded some that were still ongoing and the results were not yet reported. Thus, further studies are warranted to assess the efficacy of ADCs for treatment of HER2-positive advanced BC. Second, the safety of ADCs was not evaluated in detail. But the results of priori on the original studies showed that the ADCs group had a lower incidence of adverse events compared with other HER2-targeted therapies. Third, the study lacked stratified analysis on sex, age, region, and drugs used in adjuvant treatment stage due to the unavailability of relevant experimental data. Hence, more detailed baseline data should be provided in future studies. Finally, one of the included studies only provided 97.5% CIs, so 95% CIs were calculated, which could have introduced bias.

## Conclusion

The results of this meta-analysis revealed superior therapeutic efficacy of ADCs as compared to other HER2-targeted regimens for treatment of HER2-positive locally advanced or metastatic BC, especially for patients who previously received HER2-targeted therapy, as both PFS and OS were significantly improved. These findings provide a theoretical basis for evidence-based clinical decisions.

### Electronic supplementary material

Below is the link to the electronic supplementary material.

Supplementary Material 1

## Data Availability

The original datasets for this study are included in the article/Supplementary Material.
