# Supplementary material for: Relative efficacy of antibody-drug conjugates and other anti-HER2 treatments on survival in HER2-positive advanced breast cancer: a systematic review and meta-analysis
Source: BMC Cancer. 2024 Jun 8;24:708. doi: 10.1186/s12885-024-12478-1 (PMC11162572; doi:10.1186/s12885-024-12478-1)
Supplement: Supplementary file 1 — Supplementary Material 1 [file 12885_2024_12478_MOESM1_ESM.docx]

| Database | Date of search | Results |
| --- | --- | --- |
| PubMed | from inception to March 2023 | 174 |
| Web Of Science | from inception to March 2023 | 560 |
| Cochrane Library | from inception to March 2023 | 55 |
| EMBASE | from inception to March 2023 | 514 |
| Clinical Trail | from inception to March 2023 | 8 |

Search strategy
